# Compact analytical flow system for the simultaneous determination of l-lactic and l-malic in red wines

**DOI:** 10.1038/s41598-020-76502-7

**Published:** 2020-11-10

**Authors:** Pablo Giménez-Gómez, Manuel Gutiérrez-Capitán, Fina Capdevila, Anna Puig-Pujol, Cecilia Jiménez-Jorquera, César Fernández-Sánchez

**Affiliations:** 1 Instituto de Microelectrónica de Barcelona (IMB-CNM), CSIC, Campus UAB, 08193 Bellaterra, Spain; 2 Institut Català de La Vinya i el Vi (IRTA-INCAVI), Plaça Àgora 2, 08720 Vilafranca del Penedès, Spain; 3 CIBER de Bioingeniería, Biomateriales y Nonomedicina (CIBER-BBN), Jordi Girona 18-26, 08034 Barcelona, Spain

**Keywords:** Lab-on-a-chip, Microfluidics, Sensors, Bioanalytical chemistry, Electrochemistry

## Abstract

During the malolactic fermentation of red wines, l-malic acid is mainly converted to l-lactic acid. Both acids should be precisely measured during the entire process to guarantee the quality of the final wine, thus making real-time monitoring approaches of great importance in the winemaking industry. Traditional analytical methods based on laboratory procedures are currently applied and cannot be deployed on-site. In this work, we report on the design and development of a bi-parametric compact analytical flow system integrating two electrochemical biosensors that could be potentially applied in this scenario. The developed flow-system will allow for the first time the simultaneous measurement of both acids in real scenarios at the real-time and in remote way. Miniaturized thin-film platinum four-electrode chips are fabricated on silicon substrates by standard photolithographic techniques and further implemented in a polymeric fluidic structure. This includes a 15 µL flow cell together with the required fluidic channels for sample and reagent fluid management. The four-electrode chip includes counter and pseudo-reference electrodes together with two working electrodes. These are sequentially modified with electropolymerized polypyrrole membranes that entrap the specific receptors for selectively detecting both target analytes. The analytical performance of both biosensors is studied by chronoamperometry, showing a linear range from 5 × 10^−6^ to 1 × 10^−4^ M (LOD of 3.2 ± 0.3 × 10^−6^ M) and from 1 × 10^−7^ to 1 × 10^−6^ M (LOD of 6.7 ± 0.2 × 10^−8^ M) for the l-lactate and the l-malate, respectively. Both biosensors show long-term stability, retaining more than the 90% of their initial sensitivity after more than 30 days, this being a prerequisite for monitoring the whole process of the malolactic fermentation of the red wines (time between 20 and 40 days). The flow system performance is assessed with several wine samples collected during the malolactic fermentation process of three red wines, showing an excellent agreement with the results obtained with the standard method.

## Introduction

The malolactic fermentation (MLF) is a process in the winemaking industry in which bacteria convert l-malic acid into primarily l-lactic acid. The aroma and taste of many wines depend on this process, especially in red wines, but also in certain types of white wines. Besides, this process enables the stabilization of the wine colour, and it also allows its microbiological and bacterial control^1^. The control of the MLF during all the process (from 20 to 40 days) is crucial to obtain a highly qualified wine. Current standard methods^2^ are applied in decentralized laboratories located far away from the wineries, meaning in long processes with associated high costs. They are based on chromatography and colorimetry and used bulky equipment which has to be used by highly skilled personnel. Enzymatic approaches based on absorbance detection of nicotinamide adenine dinucleotide (NADH) have been also proposed^3,4^, but they are also applied in external laboratories, meaning in more extra steps to carry out the process (sample uptake, sample stabilisation, sample transport and sample storage). This challenge should be solved for enabling on-time and in-situ corrective actions in the MLF process, to correct possible unpredictable problems.

The miniaturization of analytical methods could be of high interest for this type of applications because they enable the integration of multiplexed analysis in low-cost and fast-response portable devices by requiring very low volume of reagents^5^. Compact and portable flow-systems should have an associated manufacturing and maintenance low cost to be competitive in the winemaking industry. Besides, the materials used to fabricate them have to be cheap and tough, but also easy to machine. First approaches done in research for these devices used glass, ceramic and silicon^6^ because they are easy to manufacture and very reproducible, but the integration of sensors or other flow elements is very complicated. To solve this challenge, polymers have been proposed from several decades ago for flow-systems because they are very low cost^7^. The most common polymer used in fluidic systems is the polymethyl methacrylate (PMMA)^8^ because it is rigid, hard and very easy to manufacture by fast-prototyping techniques (i.e. milling and laser ablation)^9–14^. Regarding the sensing part of the device, the electrochemical biosensors have been extensively reported as the best chance for the monitoring of analytical processes, including food control. They are also easy to integrate in portable analytical flow-systems for on-site analysis^15^.

We previously reported on the development of individual amperometric biosensors for the detection of l-lactate and l-malate in batch. Both biosensors showed long-term working stability of more than 37 days. This is the key factor that enabled the application of these biosensors to monitor the MLF process. The biosensors’ architecture included a thin-film electrochemical transducer selectively electromodified with a bienzymatic membrane, based on a three-dimensional matrix of electrogenerated polypyrrole (PPy). For the l-lactate biosensor (Fig. S1a, in the Supplementary Information-SI), the PPy membrane entrapped lactate oxidase (LOX) and horseradish peroxidase (HRP) as enzymes, while ferrocyanide (Fe(CN)_6_^4-^) in solution was used as redox mediator^16^. In the case of the l-malate biosensor (Fig. S1b, in the SI), the PPy membrane entrapped malate dehydrogenase (MDH) and diaphorase (DP) as enzymes, together with hexaammineruthenium (III) (HAR) as redox mediator, while β-Nicotinamide adenine dinucleotide (NAD^+^) in solution was used as co-factor^17^. In both cases, the selected redox media was oxidized or reduced on the surface of the electrochemical transducer, and the faradic signal resulted from this process was associated to the concentration of the analyte in the sample.

In this work, the production of a miniaturized flow-system integrating the above described biosensors is addressed for the very first time. The developed cost-effective flow-system could be readily used in field and in an automatic fashion, representing a significant advance in the precise monitoring of the malolactic fermentation in the winemaking industry. The simultaneous determination of l-lactic and l-malic in red wine samples with this flow-system is thoroughly assessed. A silicon chip comprising a platinum four-electrode electrochemical cell (counter, pseudo-reference and two working electrodes) was integrated in the compact flow-system. The working electrodes were selectively and sequentially modified with electrogenerated polypyrrole membranes to construct the corresponding on-chip biosensors. The resulting biochip was integrated in a multi-layered PMMA flow cell fabricated by a laser cutting process, which allowed the simple alignment of the chip with the fluidic reservoir and channels. Then, the bi-parametric fluidic system was applied to the monitoring of l-lactic and l-malic in samples collected along the MLF process of three different red wines and the results were compared with those recorded with the standard methods.

## Experimental

### Reagents and solutions

High pure (or analytical grade) reagents from Sigma-Aldrich (Spain) were used in this work. Deionized water was used to prepare the solutions. For the mechanical cleaning of the electrodes, ethanol 96% and 6 M sulfuric acid (H_2_SO_4_) were used. The distillation of the pyrrole (reagent grade, 98%) was carried out once per week and then it was preserved at − 20 °C in the freezer. Potassium phosphate monobasic (KH_2_PO_4_) was used to prepare a 0.05 M phosphate buffer (PB) solution (pH 7), which was used for the sensors fabrication and their characterization.

The bienzymatic l-lactate biosensor was fabricated by using 10-µL aliquots of 1 U µL^−1^ Lactate oxidase (LOX, from *Pediococcus* sp., lyophilized powder, ≥ 20 U mg^−1^ solid), and then they were preserved at − 20 °C. The enzyme horseradish peroxidase (HRP, type VI-A, essentially salt-free, lyophilized powder, 250–330 U mg^−1^ solid) was preserved in a refrigerator at 4 °C as it was purchased. As redox mediator for the HRP enzyme, the potassium ferrocyanide (K_4_[Fe(CN)_6_]) was employed. The bienzymatic l-malate biosensor was fabricated by using 15-µL aliquots of 5 U µL^−1^ Malate dehydrogenase (MDH, from porcine heart, freeze-dried material, ≥ 119 U mg^−1^ solid, Sorachim, S.A.) preserved at − 20 °C. β-Nicotinamide adenine dinucleotide hydrate (NAD^+^, ≥ 96.5% enzymatic, from yeast) and diaphorase (DP, from *Clostridium kluyveri*, lyophilized powder, 3–20 U mg^−1^ solid) were preserved in a freezer at − 20 °C as they were purchased. Every day, a 1-mL solution containing 0.1 M NAD^+^ was prepared to be used as co-factor for the MDH. Moreover, the reagent hexaammineruthenium(III) chloride (Ru(NH_3_)_6_Cl_3_, 98%) (HAR) was used as redox mediator for the DP.

### Devices and equipment

A 11 × 9-mm^2^ silicon chip formed by four in-parallel platinum (Pt) microelectrodes was designed and fabricated by using standard photolithographic techniques^18^ (Fig. S2, in SI). The larger electrode (2 × 2.5 mm^2^) was used as counter electrode (CE), the two internal ones (1 × 2.5 mm^2^) were used as working electrodes (WE 1 and WE 2) and the last one (1 × 2.5 mm^2^) was used as pseudo-reference electrode (p-RE). The separation between adjacent electrodes was 0.6 mm and the contact pads were located 2.9 mm far from the electrode areas.

**Figure 1:**
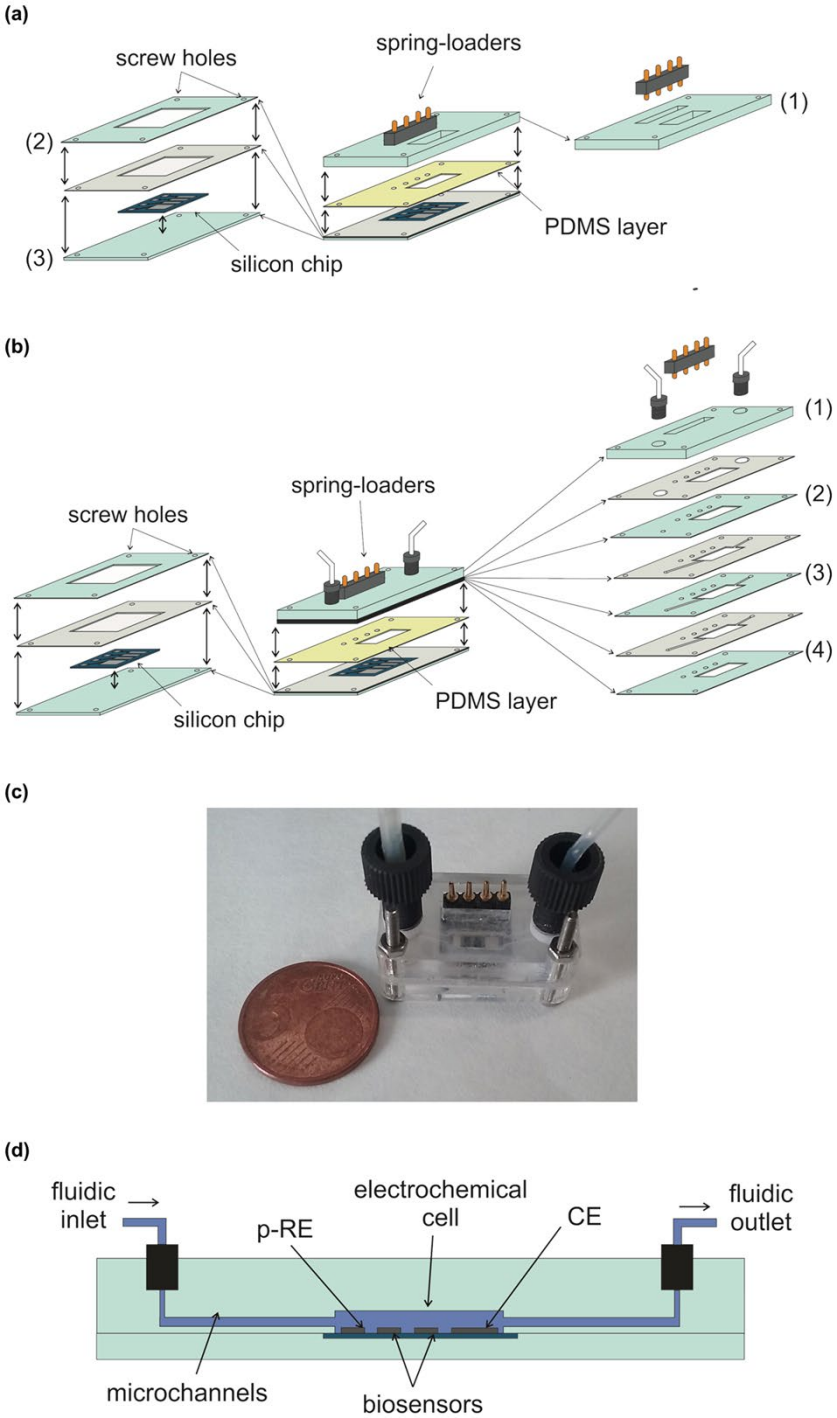
**a** Scheme of the PMMA cell used for the biosensors fabrication: layer 1 defines the 50-µL electrochemical cell, and layers 2 and 3 define the well to host and align the silicon chip and the electrical connector. **b** Scheme of the PMMA cell used for the biosensors characterization: layer 1–4 define the 15-µL electrochemical cell, the fluidic channels and the well to host the electrical connector. **c** Image of the assembled flow-system used for the biosensors characterization. **d** Scheme of the cross-section of the bi-parametric compact analytical flow-system detailing the fluidic performance inside the PMMA structure used for the biosensors characterization.

The chip was inserted in a PMMA cell, designed by Corel Draw v.17 software and machined using a CO_2_-laser printer (Epilog Mini 24, Epilog Laser, USA). Two different cell architectures were mechanized. The first one, shown in Fig. 1a, was used for the batch sequential fabrication of both sensors, whereas the second one, shown in Fig. 1b, was used for the sensor analytical characterization under flow conditions. The chip can be used without any encapsulation process and was directly inserted in the cell where an integrated four spring-loaded connector (RS Components, Switzerland) was placed in contact with the electrode pads to enable contacting the chip with the measuring instrument. The distance from the electrodes to the contact pads enabled leaving enough room for the fluidic cell, providing an easy integration of the chip into the flow cell and a proper approach for connecting the cell to the measuring potentiostatic device. Both PMMA cells were formed by a 3-mm-thick PMMA bottom part fixed to a 0.5-mm-thick PMMA layer using double-sided PSA (175 μm thick) as adhesive. The 0.5-mm layer defined a 11 × 9 × 0.5-mm^3^ well to host and align the silicon chip. The top PMMA part was 5-mm-thick. The one used for the sensor fabrication (Fig. 1a) defined a 50-µL chamber, which was aligned over the area of the electrodes. A 180-µm-thick PDMS layer was sandwiched between the bottom and top PMMA layers and the three layers were clamped together with four 1-mm diameter screws to avoid fluid leakage. During all the electropolymerization and activation steps, a 2-mm-diameter stainless-steel wire was used as CE and a 1.5-mm-diameter Ag/AgCl (3 M KCl) flexible Dri-Ref (World Precision Instruments, Sarasota, USA) was used as RE.

The top PMMA part of the flow cell used for the sensor characterization (Fig. 1b) included several layers, which were fixed using 175-µm-thick double-sided PSA as adhesive. It comprised a 15-µL cell, two fluidic channels (1 mm width, 7 mm length) showing a thickness of 175 μm and 1-mm-diameter holes to enable fluidic connection between consecutive layers. Two fitting threads for connecting the fluidic inlet and outlet with external Teflon tubes (1.0-mm inner diameter, Teknokroma, Barcelona, Spain) were also included. A 180-µm-thick PDMS layer was also sandwiched between the top and bottom PMMA parts and fixed with four 1-mm diameter screws to avoid the fluid leakage. Here, the electrochemical cell comprised both biosensors together with the integrated Pt on-chip CE and p-RE electrodes. An image of the assembled compact analytical flow-system is shown in Fig. 1c. A cross-section of the fabricated bi-parametric compact analytical flow-system is shown in Fig. 1d. Conditioned samples were flowed inside the device by using a peristaltic pump (403U/VM3, Watson Marlow, UK) in this first approach.

An Autolab workstation (PGSTAT-100 potentiostat—galvanostat, Ecochemie, Uthecht, The Netherlands) was employed to carry out the electrochemical measurements. The potentiostat was controlled by using the software NOVA v2.0 (Metrohm Autolab, Utrecht, Netherlands).

### Electrochemical procedures

Firstly, the electrodes were cleaned and activated as follows: 96% ethanol, 6 M H_2_SO_4_ and deionized water were used to mechanically clean the surface of the electrodes, and subsequently they were electrochemically activated by cyclic voltammetry (20 scans from + 0.8 to − 2.2 V at 100 mV s^−1^) in a 0.1 M KNO_3_ solution^18^. Then, the surface of both WEs was selectively electro-modified by applying the conditions optimized in our previous works, allowing the integration of the two biosensors on a single chip. A l-lactate biosensor was constructed in the WE 1 and a l-malate biosensor in the WE 2. In both cases, the PPy membranes were electrogenerated by fixing an overpotential of + 0.7 V (vs. Ag/AgCl) in a 50-μL 0.05 M PB solution at pH 7, which also contained 0.4 M pyrrole and 0.1 M KCl (named after generation solution). For the fabrication of the l-lactate biosensor, the generation solution also included 10 U of LOX and 200 U of HRP. The overpotential was applied until reach an accumulation charge of 500 mC cm^−2^^16^. For the electrosynthesis of the l-malate biosensor, a first membrane of PPy with an accumulated charge of 250-mC cm^−2^ was electrosynthesized. The generation solution used in this step also included 10 mM HAR(III) as redox mediator. Then, a second membrane of PPy with an accumulated charge of 500 mC cm^−2^ was generated by adding to the generation solution 45 U of MDH and 7.5 U of DP^17^. Afterward, the as-produced two-biosensor chips were rinsed with PB solution to remove the (bio)reagents physically adsorbed onto the PPy surface. Finally, they were preserved in a freezer at 4 °C in a PB solution when they were not in use. For ensuring a stable base line for both biosensors through all the measurements, the PPy membranes were overoxidized just after their electrosynthesis. This oversoxidation was carried out by cycling the potential from 0 to + 1 V 60 times at 100 mV s^−1^ in a PB solution^19^. The overoxidation process only had to be done once after the electrosynthesis of the biosensors, and it was not necessary to repeat the process during the life of the biosensors.

The biosensor responses were based on the cascade (bio) reactions depicted in Fig. S1 (in the SI). All analytical measurements were carried out in the electrochemical flow cell and under stop flow conditions. The p-RE was positioned upstream the biosensors to avoid the potential changes caused by the enzymatic reactions on the biosensors. Initially, cyclic voltammograms (CVs) were recorded at 20 mV s^−1^ by injecting 125 μL of a 0.05 M PB solution (pH 7) containing 0.5 M KCl and all the other reagents required to complete the bi-enzymatic reaction for both biosensors. For the l-lactate biosensor, 1 mM l-lactate and 2 mM K_4_[Fe(CN)_6_] as redox mediator were added. Regarding the l-malate biosensor, 1 mM l-malate and 5 mM NAD^+^ as co-factor were added to the characterization solution. The solution also included 0.5 M KCl to minimize the potential drop and the hysteresis effects of the electrochemical processes, which were observed when biosensors were measured in compact cells under flow conditions^20^. Once the optimum operational potential applied for chronoamperometric measurements was set, calibration curves were performed in triplicate for both target analytes. l-lactate was measured in a concentration range between 1 × 10^–7^ and 1 × 10^–3^ M, whereas l-malate was measured in a concentration range between 1 × 10^–7^ and 1 × 10^–5^ M.

The biosensors’ performance was assessed in terms of sensitivity, linear range, limit of detection (LOD) and reproducibility of the fabrication procedure by using three biosensors fabricated under the same experimental conditions. Biosensor selectivity was evaluated in PB solutions containing the main interferences in wine samples (glycerol, glucose, gluconic acid, fructose, acetic acid, citric acid, ethanol, l-lactic, l-malic, tartaric acid and ascorbic acid) with a concentration of 5 × 10^–5^ M or 5 × 10^–7^ M for the l-lactate and the l-malate detection, respectively. A set potential of 0.35 V and − 0.4 V (vs Pt p-RE) was applied for the l-lactate and the l-malate detection, respectively. Concerning the working stability with time over long-times, both bio-chips were tested by calibrating them every 2 or 4 days in a concentration range of 1 × 10^–7^ M–1 × 10^–5^ M and 1 × 10^–6^ M–1 × 10^–4^ M for the l-malate and the l-lactate, respectively.

All solutions and samples used for the evaluation of the bi-parametric compact analytical flow-system were flowed continuously during 30 s at 0.25 mL min^−1^, in order to ensure that the previous solution filling the electrochemical flow cell was flowed out and replaced by fresh solution for carrying out the following measurement.

### Red wine samples from malolactic fermentation

Three different red wines provided by the Catalan Institute of Vineyard and Wine (IRTA-INCAVI) were selected to validate the developed flow-system for the simultaneous determination of l-malic and l-lactic. The wines were collected from the 2013 vintage and their vineyards were harvested in the region of Tarragona (Spain). The MLF process was induced in the samples after the alcoholic fermentation process by a strain of the specie *Oenococcus oeni*. A set of samples for each of the three wines were collected along the MLF process, in a concentration range from 0 to 8 × 10^–3^ M (0–1.2 g L^−1^) for the l-malic, and from 0 to 6 × 10^–3^ M (0–0.5 g L^−1^) for the l-lactic acid. The number of the collected samples was 10 of the Wine 1 (along 28 days), 5 of the Wine 2 (along 33 days) and 13 of the Wine 3 (along 45 days). All the samples were analysed with the bi-parametric compact analytical flow-system. The wine samples were diluted to adjust the l-malic acid and the l-lactic acid concentrations to the linear range of the individual biosensors obtained previously in our group (from 5 × 10^–6^ to 1 × 10^–4^ M and from 1 × 10^–7^ to 1 × 10^–6^ M, for the l-lactate and the l-malate, respectively)^14,15^. The wine samples were eventually diluted 1:10,000 by carrying out two intermediate dilutions of 1:100 in a 0.05 M PB solution (pH 7) containing 0.5 M KCl to adjust the l-malic acid concentration to the linear range of the biosensor. For the l-lactic determination, the dilution in a PB solution to adjust the concentration to the linear range of the biosensor for Wine 1 and Wine 2 was 1:50, meanwhile for Wine 3 was 1:20.

The results obtained with the bi-parametric compact analytical flow-system were compared with them obtained by the standard enzymatic method applied by the IRTA-INCAVI. The standard method is based on the enzymatically catalysed reaction between the l-lactate or l-malate and the NAD^+^ to produce NADH, whose concentration is stoichiometrically related to the analyte concentration in the sample. The change of the NADH concentration is measured spectrophotometrically at 340 nm^21^.

Regarding the hydrodynamic performance of the bi-parametric compact analytical flow-system, the procedure used for the detection of both parameters is summarized as follows: Firstly, the wine sample was diluted in two 125-μL PB solutions for adjusting the concentration of the parameter to the linear range of each biosensor. Then, for the l-lactic detection, the ferrocyanide was added to one of these diluted solutions and was inoculated in the bi-parametric compact analytical flow-system during 30 s to totally clean the system from the last sample. The same procedure was repeated for the l-malic detection, but inoculating the diluted sample containing the NAD^+^.

## Results

### Biosensor fabrication

**Figure 2:**
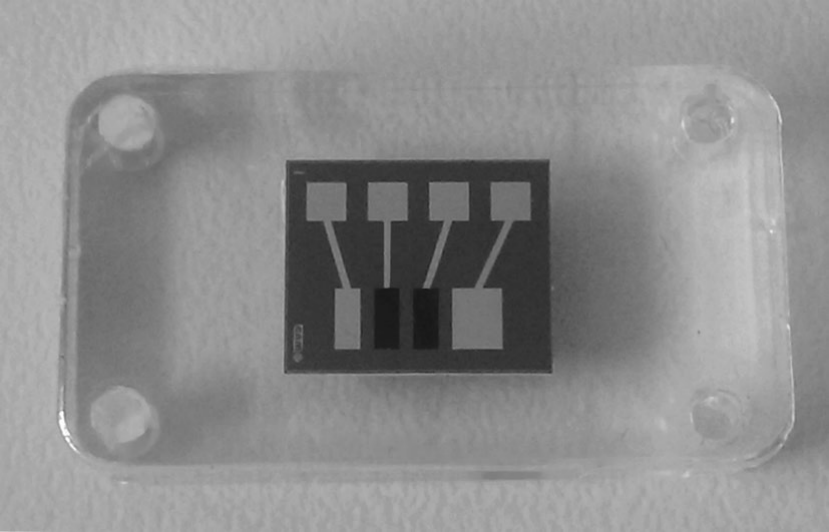
Image of the amperometric platinum microelectrode showing the polymer films selectively deposited on the surface of both working electrodes.

The use of electropolymerized membranes to produce the biosensor recognition layers on top of the electrodes allowed for the on-chip integration of both l-lactate and l-malate biosensors. The electrosynthesis of both PPy membranes was sequentially carried out in the electrochemical flow cell by applying potentiostatic conditions, as was described in the experimental section. The electrogeneration method applied avoided the cross contamination between the four integrated electrodes of the silicon chip. As can be seen in Fig. 2, both biosensors were selectively electrodeposited in the WE 1 and WE 2. The current profiles recorded under potentiostatic conditions during the potentiostatic electropolymerization of the PPy films are depicted in Fig. S3 (in the SI). The chip architecture facilitates the integration in a flow cell and can easily be connected via the embedded spring-loaded connectors to the measuring instrument. Millimetre-sized electrodes show suitable dimensions to work in a compact flow system like the one shown in this work with the electrode distance and electrochemical cell layout being adequate to avoid any electrochemical cross-talk due to the reactions taking place at the counter electrode on the biosensor devices and Ohmic drop. The biosensor fabrication was carried out sequentially without observing any chemical interference. The pseudo-reference electrode was placed upstream the biosensors to avoid any potential changes caused by the enzymatic reaction. Also, the counter electrode was placed downstream to avoid possible interference on the biosensor responses, as pointed out above. No chemical cross-reactions were observed in the biosensor responses, convincingly demonstrating the suitability of the architecture used to develop the chip.

In both biosensors, the PPy membranes were overoxidized in order to obtain a stable base-line signal. This process consisted of carrying out 60 consecutive potential cycles between 0 and + 1 V at 100 mV s^−1^ in a 0.05 M PB solution (pH 7)^17^. The voltammetric signals (cycles 5, 30, 45 and 60) obtained during the overoxidation of the l-lactate and the l-malate biosensors are shown in Figs. S4a and S4b (in the SI), respectively. As can be observed, the non-faradaic current decreased with the increase in the number of CV until almost stabilization. This is associated with the electrochemical oxidative degradation of the conducting polymer^22^. Although the overoxidation of the polypyrrole membrane causes a loss of its conductivity or electroactivity, the moderate conditions applied in this work enabled to obtain a more stable background signal, which resulted in an improved analytical biosensor performance^23^.

### Analytical characterization of the bi-parametric compact analytical flow-system

The voltammetric responses recorded with the two on-chip biosensors in the electrochemical flow cell were used to fix the applied potential for the chronoamperometric characterisation of both biosensors. In the case of the l-lactate biosensor, after the addition of 1 mM l-lactate to a 0.05 M PB solution at pH 7 which also contained 0.5 KCl, an increase of the cathodic current at around − 0.35 V (vs Pt p-RE) occurred (Fig. S5a—in the SI). This increase was caused by the ferricyanide reduction, which was previously generated by the bienzymatic reaction involving the LOX and the HRP. For the l-malate biosensor, the addition of 1 mM l-malate and 5 mM NAD^+^ to a 0.05 M PB solution (pH 7) containing 0.5 M KCl caused an increase of the anodic current (Fig. S5a, in the SI). This current increase was the consequence of the re-oxidation of the HAR(II) previously generated by the bienzymatic reaction involving the MDH and the DP. According to the experimental results, − 0.35 V and − 0.4 V (vs Pt p-RE) were selected as set potential for the l-lactate and the l-malate detection, respectively.

**Figure 3:**
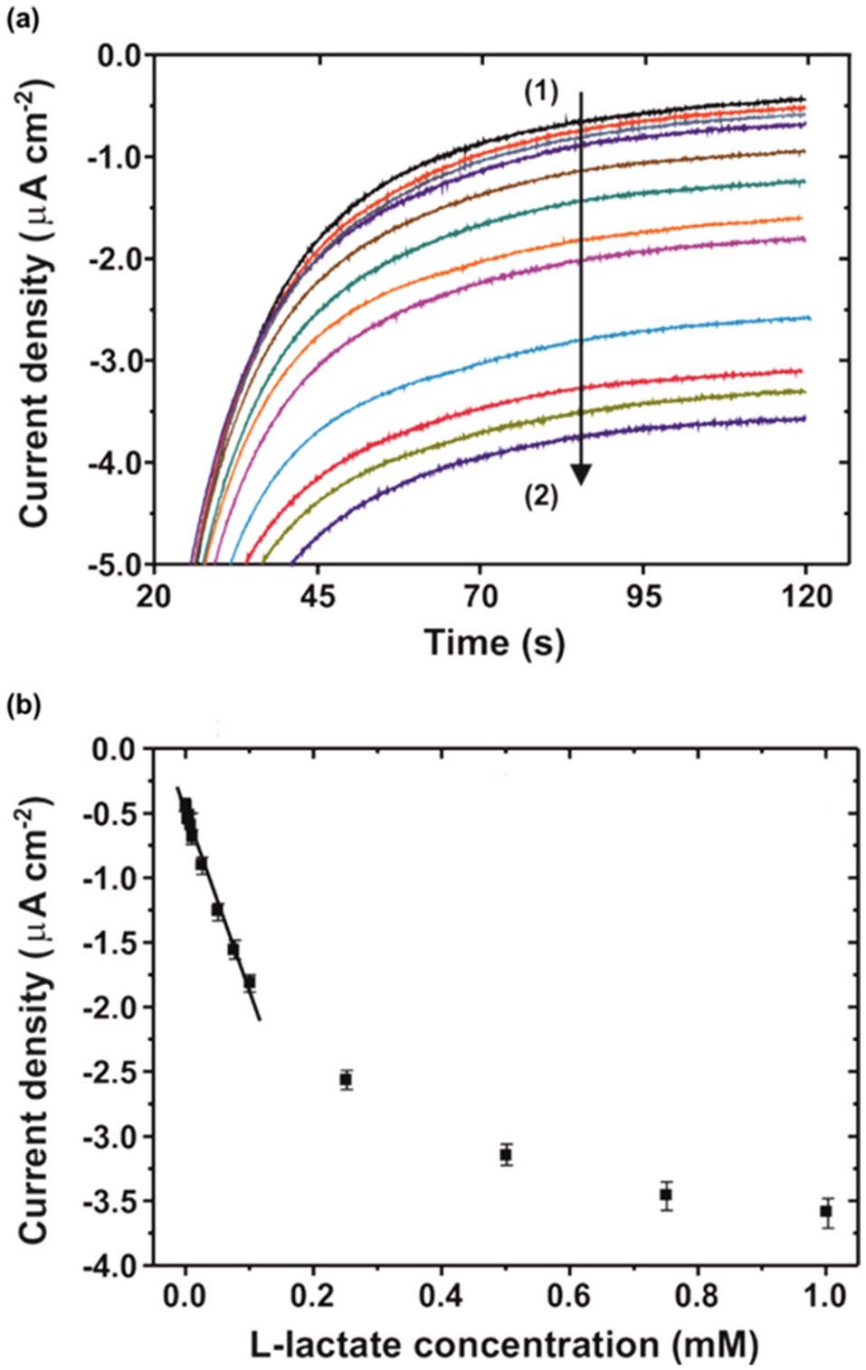
**a** Chronoamperometric curves obtained for the characterization of the l-lactate biosensor in a concentration range from (1) 1 × 10^–7^ M to (2) 1 × 10^–3^ M. **b** Calibration curve obtained from the mean value of the current density of the last 30 s for each analysed l-lactate concentration. Each point represents the mean current value of three replicates recorded consecutively with the same biosensor, with the error bars being the corresponding standard deviation.

**Figure 4:**
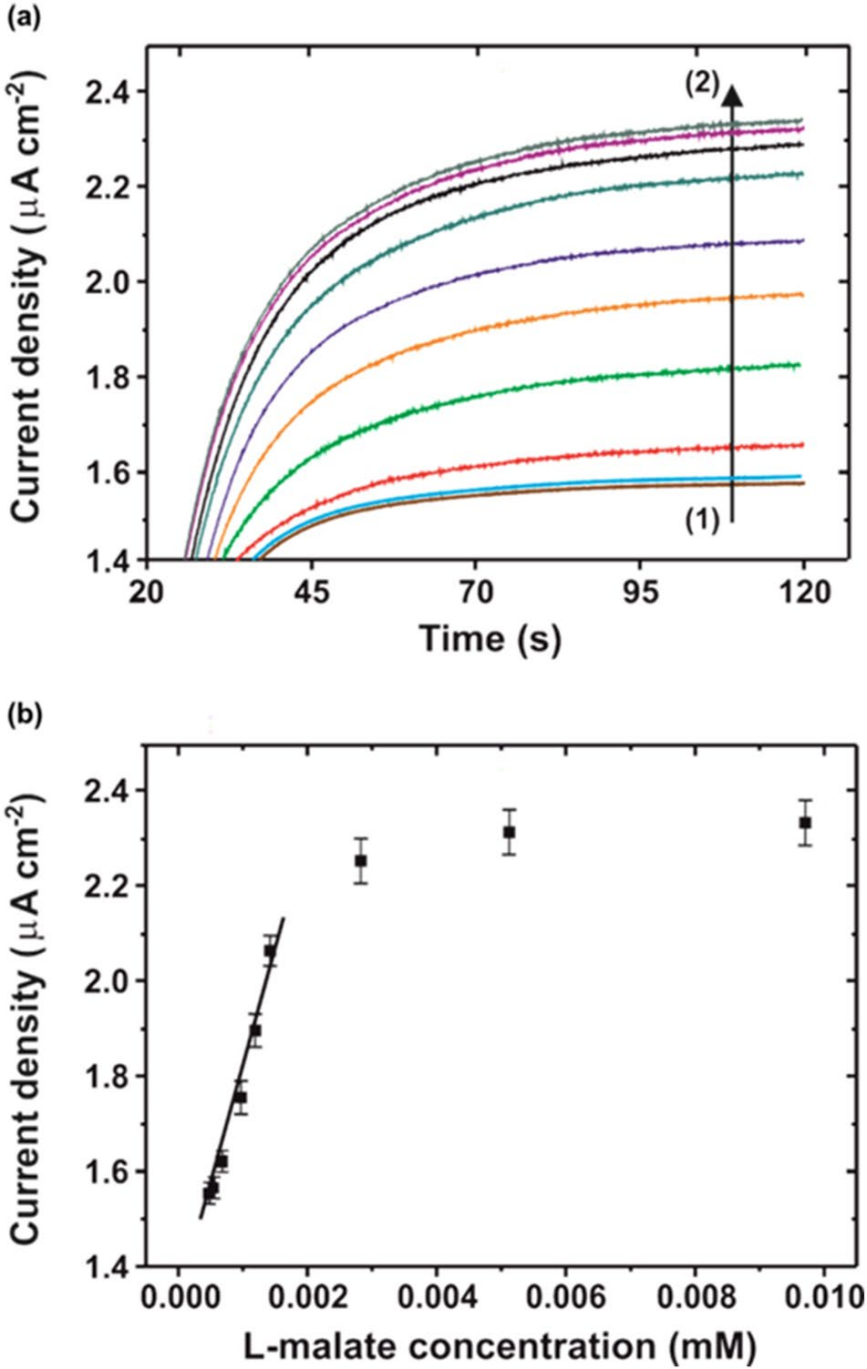
**a** Chronoamperometric curves obtained for the characterization of the l-malate biosensor in a concentration range from (1) 1 × 10^–7^ M to (2) 5 × 10^–6^ M. **b** Calibration curve obtained from the mean value of the current density of the last 30 s for each analysed l-malate concentration. Each point represents the mean current value of three replicates recorded consecutively with the same biosensor, with the error bars being the corresponding standard deviation.

From the obtained chronoamperograms in the electrochemical flow cell, the corresponding calibration curves of the two on-chip biosensors were constructed. Figure 3a shows that larger negative current densities were recorded along 120 s in solutions containing increasing concentrations of l-lactate. In all cases, the recorded currents for each analyte concentration became stabilized at around 90 s. Therefore, the mean value of the current density of the last 30 s was used as analytical signal for plotting the calibration curve for the l-lactate biosensor (Fig. 3b). Concerning the l-malate biosensor, similar response profiles were recorded but here higher anodic currents were recorded when the l-malate concentration increased in solution (Fig. 4a). The corresponding calibration curve was also plotted by using the mean current density value of the last 30 s vs. the l-malate concentration in solution (Fig. 4b). The l-lactate biosensor showed a sensitivity of (− 173 ± 8) × 10^2^ µA M^−1^ cm^−2^ (r = 0.997, n = 7) in a linear range from 5 × 10^–6^ to 1 × 10^–4^ M and a LOD (3σ IUPAC criterion) of 3.2 ± 0.3 × 10^–6^ M. A linear response for the l-malate biosensor was observed in a range from 1 × 10^–7^ to 1 × 10^–6^ M, with a sensitivity of (5.53 ± 0.6) × 10^2^ mA M^−1^ cm^−2^ (r = 0.997, n = 5) and a LOD of 6.7 ± 0.2 × 10^–8^ M. For concentrations above 1 × 10^–4^ M of l-lactate and above 1 × 10^–6^ M of l-malate, the respective enzymatic biosensors showed a saturation behaviour, this being in agreement with a process following a Michaelis–Menten kinetics. The reproducibility of the biosensors fabrication methodology was evaluated for each analyte by calibrating three different on-chip biosensors fabricated in three different silicon chips. The obtained relative standard deviation (RSD) of the biosensor sensitivity was lower than 8% and 6% for the l-lactate and the l-malate, respectively, meaning that the reproducibility of the fabrication method is very good.

The selectivity and the working stability of both biosensors were studied in detail and reported in our previous works as is detailed in the experimental section^16,17^. It can be assumed that this is going to be analogous in the bi-parametric compact analytical flow-system reported here. Regarding the selectivity, any of the checked interferences showed a signal that could affect the analyte detection at the applied set potential for both biosensors. Concerning the working stability, the biosensors maintained more than 90% of their initial response during 52 days and 37 days for the l-lactate and the l-malate detection, respectively, meaning that these integrated biosensors can be used for the monitoring of long analytical processes, such as the MLF.

### Malolactic fermentation monitoring with the bi-parametric flow-system

**Figure 5:**
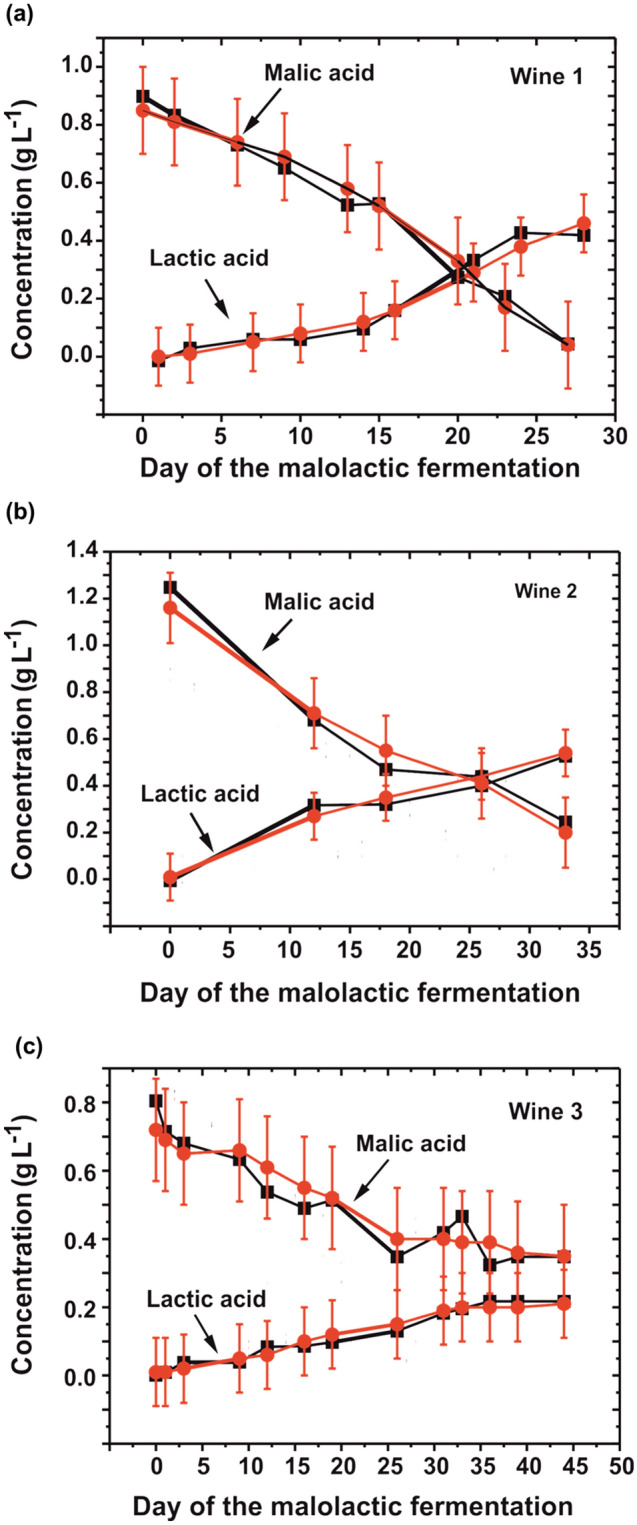
Comparative analysis of wine samples collected during the malolactic fermentation process for three red wines. Black symbols show the results of the l-lactic and l-malic acid concentration determined by the bi-parametric system. Red symbols correspond to the values obtained with the colorimetric (enzymatic) standard method. The error bars represent the uncertainty at 95% in the case of the colorimetric method.

Finally, the bi-parametric flow-system was applied to the monitoring of the malolactic fermentation of three different red wine samples collected along the MLF process. l-lactic and l-malic acids were determined consecutively in the red wine samples. Considering the required time for the electrochemical flow-cell filling (30 s) and the amperometric detection (120 s) carried out twice, the complete analysis of each sample took around 5 min. Figure 5 shows a comparison between the results obtained by the standard method and those obtained by the developed bi-parametric flow-system. There was an ideal agreement between both compared methods, with absolute errors below 0.15 g L^-1^. Moreover, it is important to notice that all the experimental values obtained with the bi-parametric flow-system are within the 95% uncertainty range of the standard method. The evolution of both acid concentrations in the samples was as expected: at the beginning of the MLF process the l-malic acid concentration was high and there was not l-lactic acid. Along the process, the concentration of l-malic acid decreased and the l-lactic acid concentration increased. Finally, the MLF process finished when both concentrations were stable. The MLF process can take from a few days to a few weeks. The control of both parameters along the MLF process is essential to ensure the quality of the wine, because it is very important to determine the end-point of the process when the complete duck-out of the l-malic acid occurred. The appropriate control of the end-point avoids the organoleptic deviations caused by the growth of damaged microorganisms, implying a new inoculation of artificial strain from laboratories for restarting the process and consequently, it provokes additional cost and time for the process. Besides, the control of l-lactic acid concentration is also necessary because the maintenance of the ratio between both acids along the process determines that there are no unwanted parallel processes in the MLF. Only one bi-parametric fluidic system without re-generating the on-chip biosensors was employed during all the assays (including the calibrations of the system performed before and after the analysis of each wine), resulting in a total number of assays above 80. It was shown that both biosensors kept more than 91% and 93% of the initial sensitivity for the l-lactate and the l-malate, respectively.

The proposed bi-parametric compact analytical flow-system reduces the number of reagents involved in the determination of both acids compared to the commercial enzymatic methods, because almost all the reagents are immobilized on the biosensor membrane, which only has to be re-generated after 37 days of intensive use. The volume of the reagents consumed is small, leading to less expense associated with the fermentation control. Moreover, the use of microfluidics enables to high dilute the wine samples, avoiding the potential matrix effects in the p-RE that could interfere in the determination of both acids. The flow-system also enables the fast and easy recalibration of the bio-chips before each analysis, meaning in a stable medium along all the assays. It is important to highlight that, in general, along the malolactic fermentation process, the l-malic acid concentration can decrease from around 3 g L^−1^ (2.2 × 10^–2^ M) to 0 g L^−1^ (0 M). Nevertheless a concentration of l-malic below 0.3 g L^−1^ (2.2 × 10^–3^ M) is considered as the end point of the MLF process in the winemaking industry. Therefore, the l-malic range can be actually considered from 2.2 × 10^–2^ to 2.2 × 10^–3^ M. Regarding the l-lactic acid, its concentration usually can increase from 0 g L^−1^ (0 M) up to 1.5 g L^−1^ (0.01 M). Thereupon, considering the general concentration ranges of these two biomarkers in red wines (from 2.2 × 10^–3^ to 2.2 × 10^–2^ M and from 0 to 1 × 10^–2^ M, for the l-malic and the l-lactic, respectively) we could anticipate that the dilution factors would be between 1000 and 10,000 for all the possible acid concentrations in the winemaking industry. For the analyses of l-lactic, a sample dilution of 1:1000 will be carried out, whereas for the analyses of l-malic the sample will be diluted 1:10,000, in order to work within the biosensor linear concentration range. In this work, a different and more adjusted dilution ratio was used for both biosensors because the concentration range during the MLF process was previously determined by the standard method, but the general dilution factor could have been applied, too.

As summary, the results in this work demonstrate the high potential of the developed compact analytical flow-system for the monitoring of both l-lactic and l-malic acids in the field of the winemaking industry. The accuracy of the proposed flow-system is appropriate for the considered application: the on-line control of both parameters in fermentation barrels of wines, which allows applying corrective actions in real time if it were necessary. Besides, the compacted size of the system will allow its easy integration within barrels, which will enable the biosensors calibration and the l-lactic and l-malic detection in an automated mode including the sample collection and dilution, the reagents inoculation, the analysis and the data processing. It results in an easier and more accurate method for the MLF process monitoring in comparison to conventional current methods without the fluidic part. Regarding the calibration of the system and the analysis of the real samples in the field, it will be as follows: the fluidic inlet of the compact analytical flow system could be easily connected to an automated and remotely controlled system of multi-valves, which will enable the flow of the rest of the reagents involved in the determination in an automatic mode. A total of six valves should be connected to 5 reservoirs containing (1) PB solution to dilute the sample, (2) l-lactate and (3) l-malate to calibrate the system every day, and (4) ferrocyanide and (5) NAD + to complete the bi-enzymatic reactions. The total volume consumed for every assay is very low (125 μL) and it is used for enabling the perfect renovation of the fluidic microchannels with next solutions, which avoids the memory effect of the sensors from previous assays by removing any trace of l-lactic or l-malic coming from them. From these numbers, and considering one calibration and one assay per day to control the malolactic fermentation process, if 10 mL containers for each solution were connected to the automated system, the microanalytical flow-system could work during all the malolactic fermentation process without any user intervention. Therefore, the proposed analytical flow-system will enable the control of the l-lactic and the l-malic acids on-site, creating a new portable and automated flow-system for the MLF process monitoring with breakthrough attributes.

### Comparison with other bi-parametric systems

Table 1 shows some characteristics of the developed bi-parametric compact analytical flow-system and other bi-parametric systems based on enzymes for simultaneous determination of l-lactic and l-malic in wines, previously reported. As can be seen, two systems use independent enzymatic reactors^24,25^, one for each parameter, two other systems are based on the immobilization of the enzymes in different membranes^26,27^ and another one is based on the entrapment of the enzymes in a solid composite^28^. The proposed bi-parametric compact analytical flow-system in this work is the only one constructed by simultaneously entrapping both enzymes, and even the redox mediator, in an electrogenerated PPy membrane. This fabrication methodology enables the deposition of the required species on the same silicon chip without affecting the other integrated electrodes, meaning in an excellent approach for the fabrication of flow devices integrating microtransducers. As a result of this simplification, there is a reduction of volumes consumed of reagents and samples in comparison to the other works in the literature, which is especially interesting for reducing costs along the monitoring of long-term processes, as the MLF.

**Table 1:** Bi-parametric enzymatic systems for simultaneous determination of l-lactic and l-malic in wines described in the literature.

Device	Detection (species)	Enzymes (l-lactate; l-malate)	LOD (l-lactate; l-malate)	Sampling	Working stability	Samples	References
FIA and enzyme reactors	Fluorimetry (NADH)	l-LDH; l-MDH	0.05; 0.1 mM*	4 min/sample	6 months	20 commercial wines	^24^
FIA, enzyme reactors and Clark electrode	Amperom. (O_2_)	LOX; MDH/DP	0.01; 0.05 mM*	4 min/sample	–	9 commercial wines	^25^
FIA and dialysis membrane	Fluorimetry (NADH)	l-LDH; l-MDH	0.11; 0.11 mM	4 min/sample	–	20 Spanish wines	^26^
FIA, nylon multienzymatic membranes and Clark electrode	Amperom. (O_2_)	l-LOX/d-LDH/HRP; l-MDH/HRP	0.028; 0.037 mM	4 min/sample	70% after 200–300 measurem	10 commercial wines	^27^
Graphite composite transducer	Chronoamp. (Ferricyanide)	l-LDH/DP; l-MDH/DP	0.011; 0.010 mM	3–6 min/sample	5–25 measurem	3 Italian wines	^28^
PPy bienzymatic membranes and Pt electrodes	Chronoamp. (Ferrocyanide; HAR)	LOX/HRP; MDH/DP	0.0032 mM; 0.067 µM	5 min/sample	92% after > 80 measurem	MLF of 3 Spanish wines	This work

*Limit of quantification.

All the other works use common species in solution for carrying out the detection of both target analytes: fluorimetric detection of NADH generated for l-LDH and l-MDH, amperometric detection of O_2_ consumed for LOX, HRP and MDH/DP and the chronoamperometric detection of the redox mediator ferricyanide. This is the reason why all of them are combined with a FIA system to manage the liquids, except one which is performed in batch^28^. In the bi-parametric compact analytical flow-system presented in this work, two specific redox mediators, ferrocyanide and HAR, were used for each analyte. In the case of the l-malate biosensor, the HAR is incorporated in the PPy membrane, allowing a continuous flow analysis.

The analysis time per sample is similar in all the cases, this being between 3 and 6 min. In the other hand, the system described in this work outperforms the other approaches in terms of limit of detection (LOD), especially in the case of l-malate. This may be partially related with the immobilization of the biochemical species in the conductive polypyrrole membrane synthesized under biocompatible conditions that may preserve the enzyme activity to a large extent.

A system applied to the monitoring of the malolactic fermentation must show a long-term working stability under continuous use because the fermentation process takes around 30 days. Some of the systems in Table 1 show good operational stability values as long as 6 months. The system developed in this work maintains 92% of its initial sensitivity after more than 80 measurements in continuous use, with a lifetime of 37 days, being able to monitor the entire malolactic fermentation process. Finally, all the systems have been applied to the bi-parametric determination in finished wines samples, which are commercially available. However, the bi-parametric compact analytical flow-system presented in this work is the only one that has been assessed using real samples collected during the malolactic fermentation of red wines. This compact analytical flow-system integrates for the first time both sensors on a new automated flow platform, resulting in a simply system for the malolactic fermentation monitoring. It represents a significant advance in the precise monitoring of the process in the winemaking industry because this novel research will provide for the very first time a cost-effective and easy-to-use way to determine l-lactic and l-malic on-site and on-line based on this new concept of test for winemaking control with unique and unprecedented attributes. No other previous reports are focused on the on chip integration of both l-malate and l-lactate biosensors in a flow-system that could show the potential of these devices for monitoring the malolactic fermentation of red wines in field and in an automatic fashion.

## Conclusions

A bi-parametric compact analytical flow system for the simultaneous determination of l-lactate and l-malate was designed, fabricated and optimized. The development of a bi-parametric compact system included the design and the fabrication of a 11 × 9-mm^2^ silicon chip comprising two working electrodes, together with a counter and a pseudo-reference electrode, all made of platinum. These miniaturized electrochemical sensors are cheap, highly reproducible and robust. The working electrodes were modified with polypyrrole-based enzymatic membranes to produce the two on-chip biosensors for l-lactate and l-malate target analytes. The use of an electropolymerization approach enabled the strict controlled deposition of the required enzymes over the selected electrodes.

The biochip did not require encapsulation and wire-bonding and thus allowed for the simple integration in a robust miniaturized PMMA flow cell, mechanized by using rapid prototyping techniques. The resulting bi-parametric compact analytical flow-system showed superior analytical and operational features for the determination of l-lactate and l-malate and was eventually assessed in red wine samples collected during the malolactic fermentation process, showing a good agreement with the results obtained for both analytes with the standard colorimetric methods. As summary, the overall novelty of the proposed flow-system is twofold: (1) it has the ability to fully process the sample and the reagents required for the malolactic fermentation control, by using a low-cost and robust miniaturized PMMA flow cell. The design of the flow cell reduces drastically the complexity of current traditional tests, meaning in a method with a total analysis time of 5 min and a required sample volume of 125 μL per analysis. Besides, the automated flow cell avoids the human error and the potential contamination of barrels derived from the manipulation of the sample and reagents. (2) The flow-system integrates both l-lactate and l-malate sensors in the same chip, which allows the reduction of reagents and sample consumption. Moreover, the monitoring of both parameters using a compact system is proposed for the very first time in literature, meaning in a novel system for the fast and total control of the malolactic fermentation in field, in real-time and on-site in the winemaking industry. The proposed flow-system would set a new paradigm for integration of electrochemical sensing in field by solving the limitations of current methods applied in the winemaking industry. This approach would open new opportunities for in-situ and real-time control of processes by making accessible to untrained personnel an easy handling method for fermentation monitoring. It would also facilitate better management to winemakers and the application of corrective actions, if required, allowing unprecedented time and cost savings.

## Supplementary information

Supplementary Information.
