# Supplementary material for: Compact analytical flow system for the simultaneous determination of l-lactic and l-malic in red wines
Source: Sci Rep. 2020 Nov 10;10:19404. doi: 10.1038/s41598-020-76502-7 (PMC7656249; doi:10.1038/s41598-020-76502-7)
Supplement: Supplementary file 1 — Supplementary Information. [file 41598_2020_76502_MOESM1_ESM.pdf]

COMPACT ANALYTICAL FLOW SYSTEM FOR THE SIMULTANEOUS DETERMINATION OF L–LACTIC AND L–MALIC IN RED WINES

Pablo Giménez-Gómez[*a], Manuel Gutiérrez-Capitán[a], Fina Capdevila[b], Anna Puig-Pujol[b], Cecilia Jiménez-Jorquera[a] and César Fernández-Sánchez[*a]

[a] *Instituto de Microelectrónica de Barcelona (IMB-CNM), CSIC, Campus UAB, 08193 Bellaterra, Spain*

[b] *Institut Català de la Vinya i el Vi (IRTA-INCAVI), Plaça Àgora 2, 08720 Vilafranca del Penedès, Spain*

[c] *CIBER de Bioingeniería, Biomateriales y Nonomedicina (CIBER-BBN), Jordi Girona 18-26, 08034 Barcelona, Spain*

[*] *Corresponding authors: Pablo Giménez-Gómez (e-mail: pablo.gimenez@csic.es) and César Fernández-Sánchez (e-mail: cesar.fernandez@csic.es)*

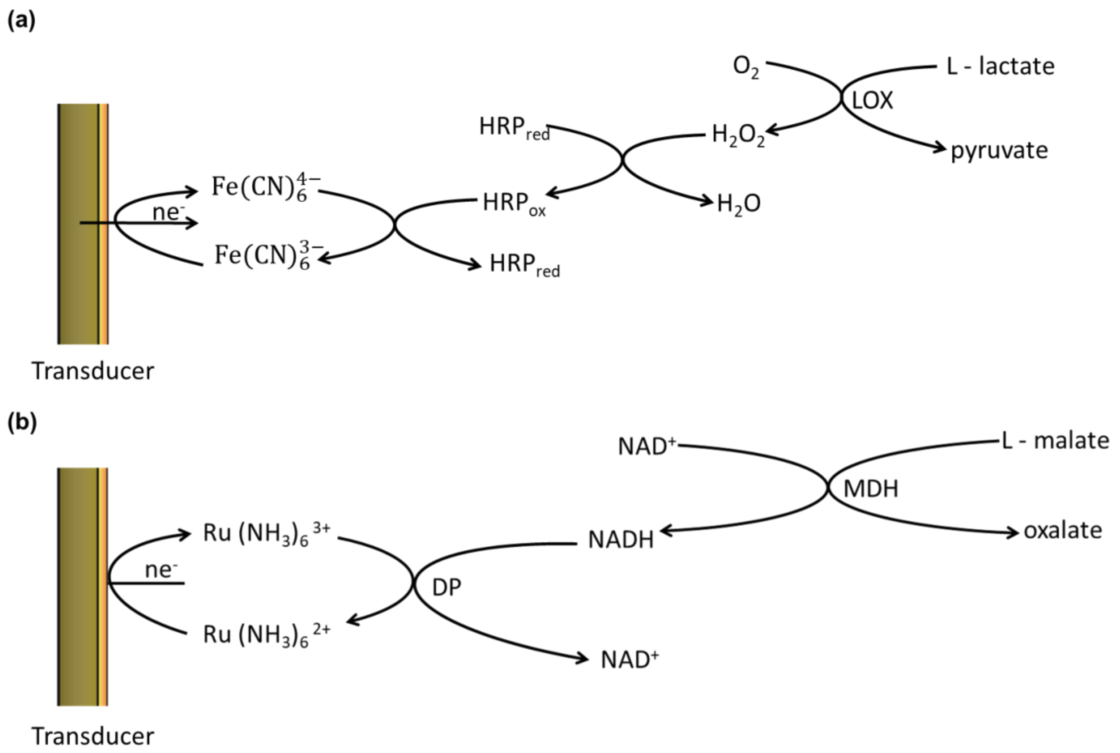

Fig. S1. Scheme of the bi–enzymatic reactions that take place during the (a) L–lactate and (b) L–malate detection.

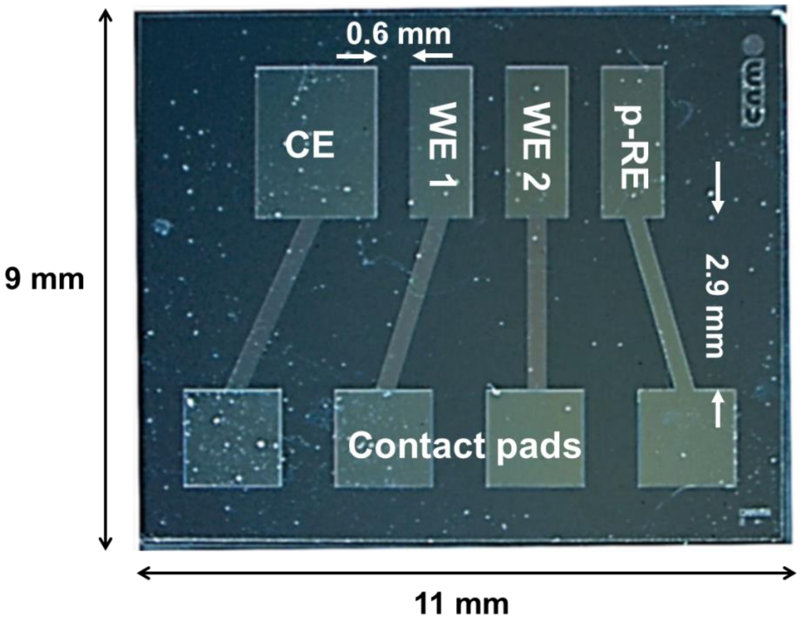

Fig. S2. Picture of the silicon chip comprising four bar–like Pt thin-film electrodes.

The current densities recorded under potentiostatic conditions during the electropolymerization of the PPy films are shown in Fig. S3. The time required for each electrosynthesis is related to the material onto which the deposition is carried out and to the composition of the generation solution. Comparing the signal for the L–lactate biosensor (black line) with the signal for the second film of the L–malate biosensor (red line), both with 500–mC cm^{-2} charge, the time needed for the L–malate (28 s) was shorter than that for the L–lactate biosensor (55 s). This is because the PPy layer grew on the surface of the first PPy membrane for the L–malate biosensor, which showed a more active area than the bare surface of the microelectrode.

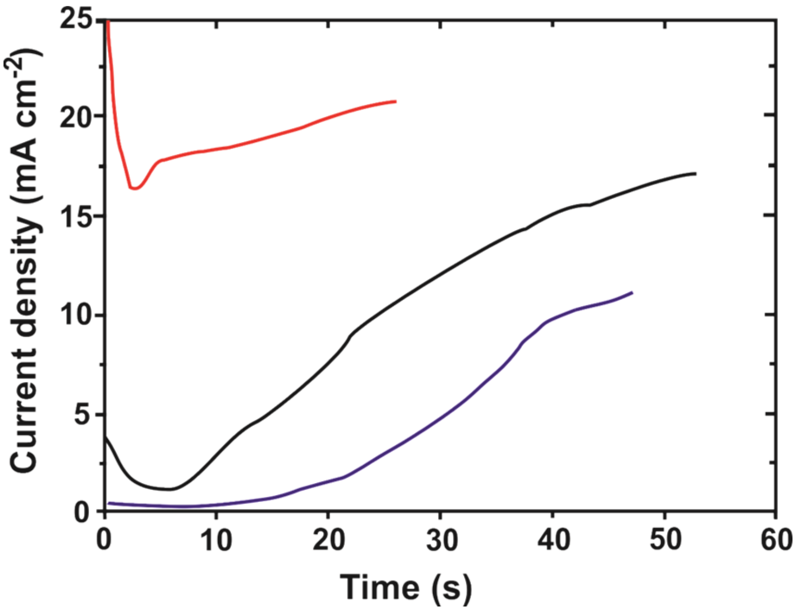

Fig. S3. Current profile recorded during the electrogeneration at +0.7 V (vs. Ag/AgCl) of the PPy films for the L–lactate biosensor (black line) and L–malate biosensor (red and blue lines). The blue corresponds to the electrogeneration of the first PPy/HAR film and the red line, to the second PPy/MDH:DP film, both of them for the L–malate biosensor.

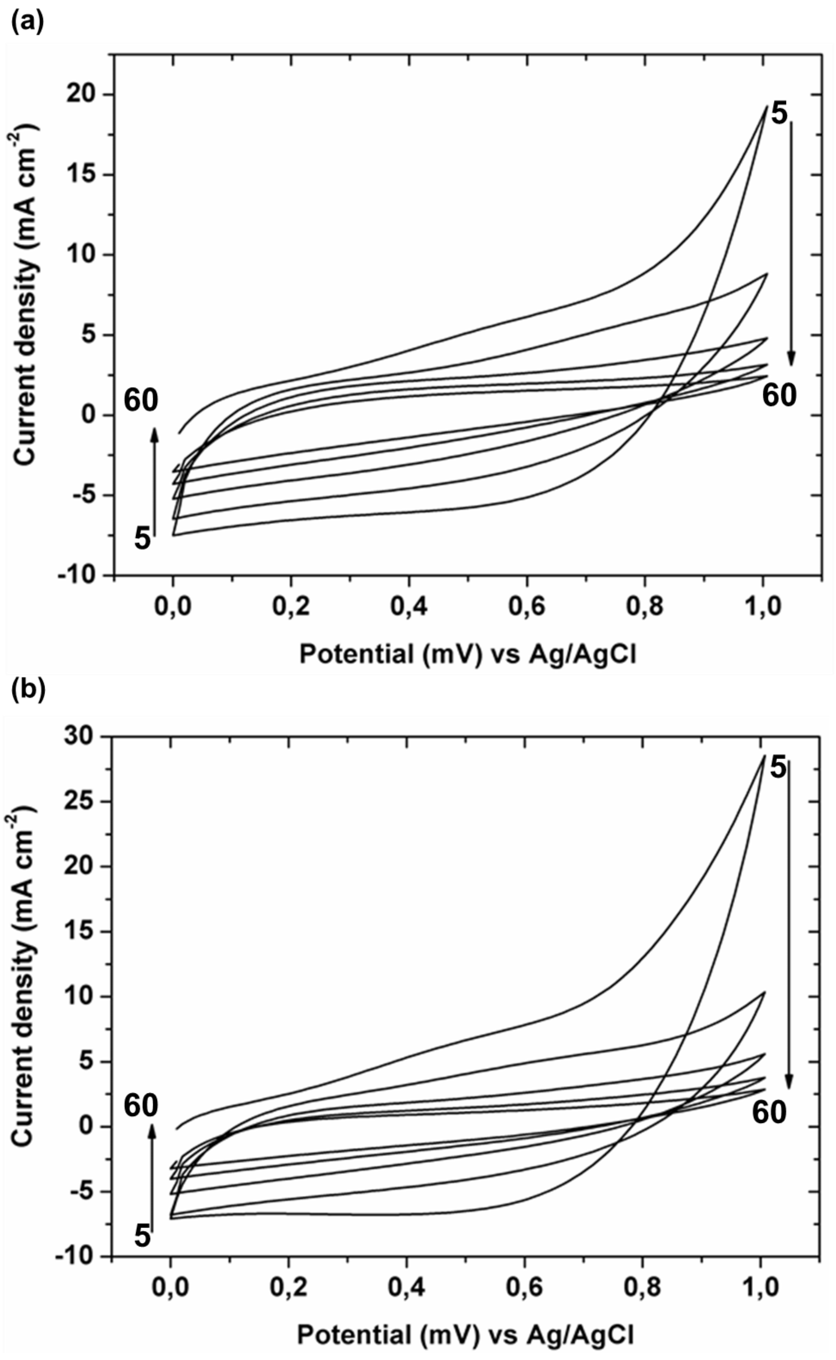

Fig. S4. Cyclic voltammograms (cycles 5, 30, 45 and 60) recorded in PB solutions during the overoxidation of the (a) L–lactate biosensor and the (b) L–malate biosensor. Scan rate: 100 mV s^{-1}

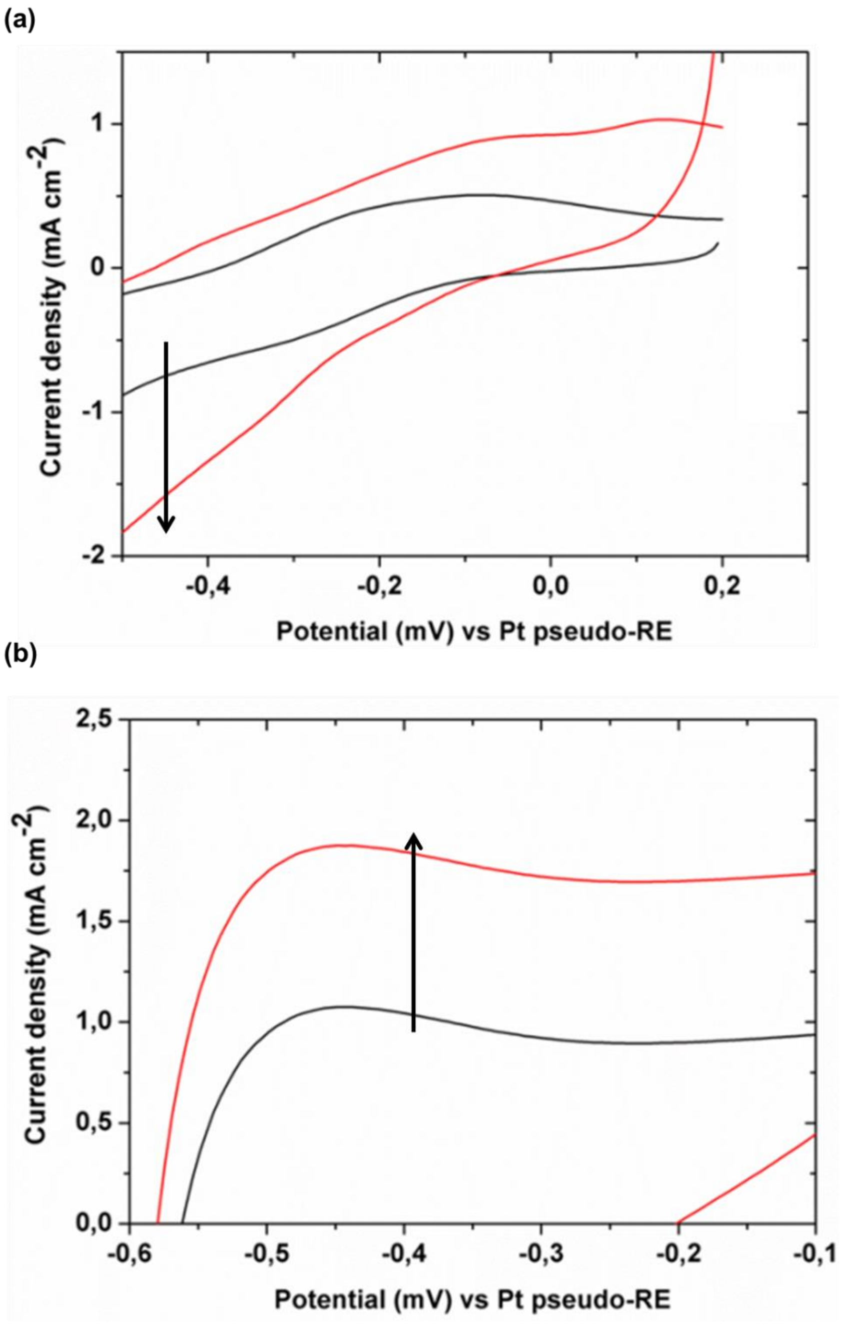

Fig. S5. Cyclic voltammograms recorded with, (a) the L–lactate and (b) the L–malate biosensor in a 0.05 M PB solution (pH 7) containing 0.5 M KCl in the absence (black line) and presence of 1 mM (red line) L–lactate and L–malate, respectively. The arrows indicate the selected potential for carrying out the subsequent chronoamperometric measurements. Scan rate: 20 mV s^{-1}
